# New-generation tetracyclines for severe macrolide-resistant Mycoplasma pneumoniae pneumonia in children: a retrospective analysis

**DOI:** 10.1186/s12879-024-10070-3

**Published:** 2024-10-16

**Authors:** Xiaoxiao Song, Ning Zhou, Shuanglong Lu, Changjuan Gu, Xiaohong Qiao

**Affiliations:** Department of Pediatrics, Tongji Hospital, School of Medicine, Tongji University, Shanghai, 200065 China

**Keywords:** Minocycline, Doxycycline, Severe macrolide-resistant *Mycoplasma pneumoniae* pneumonia

## Abstract

**Background:**

Macrolide-resistant Mycoplasma pneumoniae (MRMP) strains are increasingly prevalent, leading to a rise in severe Mycoplasma pneumoniae pneumonia incidence annually, which poses a significant threat to children’s health. This study aimed to compare the effectiveness and safety of oral minocycline and doxycycline for the treatment of severe MRMP pneumonia in children.

**Methods:**

This retrospective analysis included children treated for severe MRMP pneumonia at the Pediatric Department of Tongji Hospital, Shanghai, China, between September 2023 and January 2024 using minocycline and doxycycline. The patients were divided into four groups according to treatment: oral doxycycline alone (DOX group), oral minocycline alone (MIN group), oral doxycycline with intravenous glucocorticoids (DOXG group), and oral minocycline with intravenous glucocorticoids (MING group). Student’s *t-*test, Mann–Whitney U test, and *χ*^*2*^ or *Fisher’s* exact tests were used for group comparisons.

**Results:**

A total of 165 patients were included in this study: 84 received minocycline, and 81 received doxycycline. The DOX group had higher fever resolution rates within 24, 48, and 72 h compared to the MIN group (63.2% vs. 31.8%, 79.0% vs. 63.6%, and 100% vs. 90.9%, respectively; all *p* < 0.05). The DOXG group showed higher fever resolution rates within 24 and 48 h than the MING group (92.3% vs. 83.4%, 100% vs. 92.7%, all *p* > 0.05). There were no statistically significant differences in time to imaging improvement, cough improvement, and disappearance of wet rales between groups, regardless of glucocorticoid combination. The longer the duration of fever prior to tetracycline therapy, the greater the likelihood of hypoxemia (*p* = 0.039) and a greater than two-fold elevation in the D-dimer level (*p* = 0.004).Univariate binary logistic regression model analysis revealed that CRP and erythrocyte sedimentation rate at disease onset were associated with defervescence within 24 h after treatment with tetracyclines alone (*p* = 0.020, *p* = 0.027), with erythrocyte sedimentation rate also influencing defervescence within 48 h (*p* = 0.022).

**Conclusion:**

Doxycycline treatment resulted in a higher rate of defervescence than minocycline. Prompt treatment reduced the probability of pleural effusion, hypoxemia, pulmonary atelectasis, and D-dimer levels > 2 times the reference value.

## Background

*Mycoplasma pneumoniae* (MP) is an important pathogen that causes community-acquired pneumonia worldwide. Although most infections are mild and self-limiting, Lv et al. [1] reported that 16.0% of pediatric patients with *Mycoplasma pneumoniae* pneumonia (MPP) in Suzhou, China, from 2014 to 2020 developed severe disease. In North China, the annual incidence of severe MPP(SMPPS) increased from 0.7% in 2007 to 42.6% in 2016 [2]. This poses a significant threat to children’s health. Globally, a period of significant cyclic growth of MP at 3–5-year intervals has been reported [3], likely due to changes in the predominant circulating strains. A renewed surge in MP infections was detected in China and other countries in the fall of 2023 [4, 5].

Guidelines [6]recommend macrolides as the preferred treatment for MPP. However, macrolide-resistant Mycoplasma pneumoniae (MRMP) pneumonia has recently emerged worldwide, with the highest incidence in Asia. Approximately 80–90% of MPP cases arise in China and Japan [4], reaching 100% in China [7]. The prevalence of MRMP infections has reached 90% in Shanghai [8]. This makes the timely use of new-generation tetracyclines as alternatives to macrolide antibiotics particularly crucial. New-generation tetracyclines, including doxycycline and minocycline, can be used to treat patients with severe MRMP pneumonia. However, tetracycline therapy has potential adverse effects, including enamel hypoplasia and tooth discoloration. So far, there have been rare clinical comparative studies on the treatment of MRPP with the new generation of tetracycline. Information on their effectiveness is needed, especially in patients with severe MRPP. The objective of this study was to evaluate the rational use of new-generation tetracyclines in pediatric patients with severe MRMP pneumonia treated with minocycline or doxycycline and to use the findings to provide a source of reference on the use of new-generation tetracyclines in pediatric patients with MPP.

## Methods

### Ethics approval

This retrospective study received ethical approval from the institutional review board of ethics committee of Shanghai Tongji Hospital (Tongji University affiliated to Tongji University), (Approval No. K-W-2024-010).

The committee waived informed consent because the study was retrospective, there was no risk of harm to subjects, and all patients were anonymous.

Nevertheless, as the new-generation tetracyclines are available over the counter for children under the age of eight, it was imperative that their legal guardians be informed about the treatment and signed an informed consent form.

### Study participants

We retrospectively reviewed the clinical data of 165 patients hospitalized with severe MRMP pneumonia at the Department of Pediatrics, Tongji Hospital, Shanghai, China, from September 2023 to January 2024.

Inclusion criteria: (1) A definitive diagnosis of severe macrolide-resistant Mycoplasma pneumoniae pneumonia; (2) A comprehensive range of clinical information is available; (3) Informed consent to the use of new tetracycline antimicrobial drugs and the signing of informed consent for the use of medication by the first guardian of the patient with macrolide-resistant severe Mycoplasma pneumoniae who is less than 8 years old.

Exclusion criteria: (1) Patients received azithromycin without tetracyclines; (2) Patients with underlying diseases, such as congenital heart disease, immunodeficiency, bronchopulmonary dysplasia were excluded from the study. (3) Prior to the administration of tetracycline, the presence of additional pathogens was identified, including adenovirus, human parapneumovirus, influenza virus, parainfluenza virus, and respiratory syncytial virus, were also excluded from the study. (4) Patients who terminated the medication automatically and did not complete the course of treatment were also excluded from the study.

## Methods

### Clinical data collection

Clinical information was obtained from the hospital’s electronic case information system. Documented data included: (1) clinical information: age, sex, signs, febrile days before macrolide or doxycycline treatment, time to defervescence after macrolide or doxycycline treatment, time to cough improvement, and time to disappearance of wet rales; (2) laboratory findings: C-reactive protein, lactate dehydrogenase (LDH), cytokines, ferritin, and D-dimer levels; and (3) lung imaging: reviewed after the patient’s temperature stabilized and physical signs normalized, with the time of improvement recorded.

### Diagnostic criteria

The diagnostic criteria for community-acquired pneumonia were based on the “Community-acquired Pneumonia Diagnostic and Treatment Criteria for Children (2019 Edition)“ [9]: (1) respiratory symptoms of infection, such as fever, cough, or other extrapulmonary manifestations; (2) physical signs, mainly respiratory signs, such as shortness of breath, dyspnea, and auscultation of the lungs with wet rhonchi; and (3) imaging showing abnormal changes in the lung parenchyma or interstitial space, with or without pulmonary complications.

The diagnostic criteria for MPP and severe disease were in accordance with the “Guidelines for the Diagnosis and Treatment of *Mycoplasma pneumoniae* pneumonia in Children (2023 Edition)” [6].

For MP, at least one of the following two conditions must be met based on a diagnosis of community-acquired pneumonia: (1) a single serum MP antibody titer of ≥ 1:160 (particle agglutination); a 4-fold or greater rise in double serum MP antibody titer during the disease course; (2) Positive MP DNA or RNA results.

A severe MPP must meet any of the following manifestations: (1) Persistent high fever (above 39 ℃) for ≥ 5 d or fever lasting ≥ 7 d with no downward trend in peak temperature. (2) Wheezing, shortness of breath, dyspnea, chest pain, or hemoptysis present. (3) Development of extrapulmonary complications, not meeting critical illness criteria. (4) Finger pulse oxygen saturation ≤ 0.93 on air inhalation at rest. (5) Imaging manifestations of one of the following conditions: ① single lung lobe ≥ 2/3 involvement, the presence of uniform and consistent high-density solid lesions or two or more lung lobes with high-density solid lesions (regardless of the size of the area of involvement), possibly accompanied by moderate to large pleural effusion, or a limited manifestation of fine bronchiolitis; ② single lung diffuse or bilateral ≥ 4/5 lobes of the lung with manifestations of fine bronchiolitis, potentially combined with bronchiectasis and mucus plug formation leading to pulmonary atelectasis. (6) Progressive exacerbation of clinical symptoms and imaging showing over 50% lesion progression within 24–48 h. (7) Marked elevation of C-reactive protein (CRP), LDH, or D-dimer.

### Macrolide resistance gene test

Sputum specimens from the patients on the day of admission were retained and sent to KingMed Diagnostics Group Co., Ltd. for tNGS pathogen detection and MP mutation genotype analysis.

### Treatment

Treating severe MRMP followed the “Guidelines for the Diagnosis and Treatment of Mycoplasma pneumoniae in Children (2023 edition).” New-generation tetracyclines were administered orally to patients who showed poor response to macrolides after 72 h of regular treatment or tested positive for the macrolide resistance gene, weighing the patient’s benefit over the risk. Doxycycline hyclate enteric-coated capsules (TC Pharmaceuticals (Jiangsu) Co., Ltd., National Drug Approval No. H20030627, 0.1 g*20 capsules): the recommended dose was 2 mg/kg twice daily (q12h) orally. Minocycline hydrochloride capsules (Han Hui Pharmaceutical Co., Ltd.; National Drug Approval No. H20174081, 50 mg*20 capsules): the initial dose was 4 mg/kg, not exceeding 200 mg. The maintenance dose was given after an interval of 12 h at 2 mg/kg, twice daily (q12h) orally, with a maximum dose of 100 mg. The general course of treatment was 10d.

The following criteria were used to select new-generation tetracycline regimens during treatment:

(1). The tNGS results indicated a macrolide-resistant MP infection, which fulfilled the diagnostic criteria for SMPP. Tetracycline treatment was deemed an appropriate course of action.

(2). Following a 72-hour course of macrolide antimicrobial therapy, patients with SMPP who remain febrile and whose clinical signs and lung imaging do not improve or show further exacerbation, in conjunction with tNGS results indicating a macrolide-resistant Mycoplasma pneumoniae infection, are treated with tetracyclines.

(3). In the event that the patient is less than eight years of age, parental consent is required.

(4). The physician provides the parents with comprehensive information regarding the patient’s condition, the rationale for the use of doxycycline and minocycline, and the potential adverse effects of the medications. The parents then exercise their autonomy in selecting either doxycycline or minocycline for their child’s treatment.

Guidelines [6]recommend that patients with severe MRPP can be routinely treated with methylprednisolone. Glucocorticoids should be added to the treatment plan if the patient meets one of the following criteria: (1). High fever for ≥ 5 days (including pre-admission); (2). Significant elevation of inflammatory markers (CRP or LDH or D-dimer); (3). Imaging suggestive of a solid lung lesion, pulmonary atelectasis, or pleural effusion; (4). Rapid progression of disease (over 2–3 days). This treatment was supplemented with nebulization, expectorants, and other basic treatments.

Following the identification of the macrolide resistance gene and an assessment of the disease’s severity, a decision was made to transition these patients from macrolide therapy to a new-generation tetracyclines treatment, with the approval of the parents or legal guardians of patients under the age of eight. The tNGS results provided rapid feedback, enabling prompt adjustments to the treatment plan by the attending physicians. All children were diagnosed with severe Mycoplasma pneumoniae pneumonia, and the majority of families requested a change to new-generation tetracycline therapy following the learning of the resistance gene. A minority of children did not undergo a change in treatment regimen and were therefore excluded from the statistical analysis. It is noteworthy that some children with mild Mycoplasma pneumoniae did recover even when they continued to use azithromycin. This suggests that further data accumulation is necessary for thorough analysis and summarization.

The study was conducted in hospitalized patients with severe macrolide-resistant Mycoplasma pneumoniae pneumonia, necessitating close monitoring of their condition and response to treatment. Data collection encompassed the entire course of the illness, from the first day of symptom onset. Systematic follow-ups were conducted both during hospitalization, involving regular physical examinations and the use of standardized assessment tools, and post-discharge, through a structured schedule of physician visits at designated intervals until full recovery.

### Assessment of cough severity

Visual analog scale (VAS), a linear scale from 0 to 100 mm (0 to 100 points), was used to assess cough severity. The patient’s family members or bedside doctors marked the horizontal line daily to indicate the degree of coughing. A higher score corresponded to more severe coughing, and the treatment efficacy was assessed by a reduction of 30 mm on the VAS [10].

### Statistical analysis

Data were analyzed using SPSS 26.0 statistical software. Measurement information conforming to normal distribution was expressed as mean ± SD, and Student’s *t-*test was used for comparison between groups; non-normally distributed measurement information was expressed as median (IQR) and compared using Mann–Whitney U test. Categorical variables were expressed as the number of cases (%) of a specific group and compared using *χ*^*2*^ or *Fisher’s* exact test. Logistic regression was used to analyze the relevant factors affecting the time to fever remission after treatment with new-generation tetracyclines. Statistical significance was defined as a two-sided *p*-value of < 0.05.

## Results

### Demographic and clinical characteristics

**Fig. 1:**
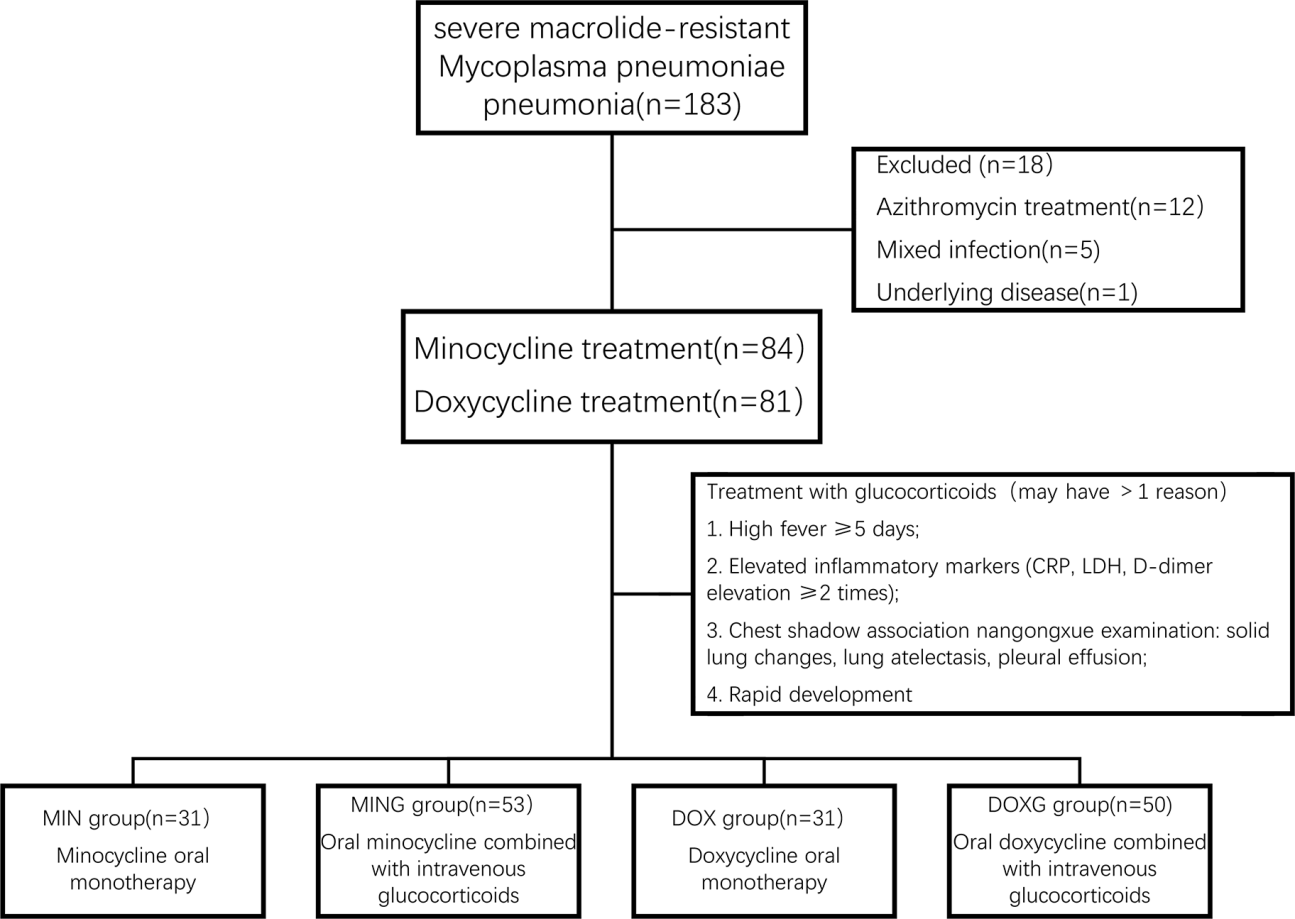
Flow diagram for the inclusion of study participants and assignment to treatment groups

This study included 165 hospitalized patients with severe MRMP pneumonia and complete clinical data from September 2023 to January 2024. Among them, 84 received minocycline treatment, with 53 (63.1%) in the MING group and 31 (36.9%) in the MIN group. The remaining 81 received doxycycline treatment, with 50 (61.7%) in the DOXG group and 31 (38.3%) in the DOX group(Fig. 1).

### Comparison of clinical characteristics between patients treated with minocycline and doxycycline

Patients treated with minocycline comprised 39 males (46.4%) and 45 females (53.6%), with a median age of 8 years (7, 9). Patients treated with doxycycline included 40 males (49.4%) and 41 females (50.6%), with a median age of 10 years (9, 11). The differences in fever duration before treatment with new-generation tetracyclines, duration of initial macrolide antimicrobials and duration of initial beta-lactam antimicrobials therapy, and number of patients receiving combined glucocorticoid therapy between the two groups were not statistically significant (Table 1). After receiving the tNGS test results, the initial antibiotic treatment regimen was switched to a new-generation tetracyclines, which required a period of 2–3 days.

All eight patients who developed hypoxemia required only nasal cannula oxygen, and all returned to a normal hypoxic state within three days. There were no instances of intubation or admission to the intensive care unit.

**Table 1:** Demographics and clinical characteristics between minocycline and doxycycline

Demographics and clinical characteristics	Minocycline (*N* = 84)	Doxycycline (*N* = 81)	*p*-value
Sex, n (%)			0.704
Male	39 (46.4%)	40 (49.4%)	
Female	45 (53.6%)	41 (50.6%)	
Age, n (%)			0.229
<6-years-old	5(6.0%)	1(1.23%)	
≥ 6-years-old	79(94.0%)	80(98.8%)	
Hypoxemia, n (%)	5 (6.0)	3 (3.7)	0.720
Fever, days, median (IQR)	4 (3, 6)	4 (3, 6)	0.614
Duration of initial antibiotic therapy, d, median (IQR)			
β-Lactam	2 (1, 3)	2 (1, 4)	0.209
Macrolides	3 (1, 4)	2 (0, 4)	0.310
Glucocorticoid, n (%)			0.856
Combination therapy	53 (63.1%)	50 (61.7%)	
Doxycycline or minocycline only	31 (36.9%)	31 (38.3%)	

IQR, interquartile range

### Comparing the clinical effectiveness of treatment with minocycline and doxycycline alone

Among the 62 patients treated with either minocycline or doxycycline, 31 were in each group. The defervescence rate within 24 h was higher in the DOX group than in the MIN group; the difference was statistically significant (*χ²* = 4.027, *p* = 0.045). The defervescence rate within 48 h and 72 h in the DOX group was also higher; however, the difference was not statistically significant (78.95% vs. 63.64%, *p* = 0.283; 100% vs. 90.91%, *Fisher* = 0.490). The median time to improvement on chest imaging was 5 d (*p* = 0.464) (Table 2).

**Table 2:** Comparison of effectiveness between the MIN group and DOX group

Parameter	MIN group	DOX group	*p* -value
Number of patients, n(%)	31(50)	31(50)	
Defervescence within 24 h, n(%)	7(31.8)	12(63.2)	0.045
Defervescence within 48 h, n(%)	14(63.6)	15(79.0)	0.283
Defervescence within 72 h, n(%)	20(90.9)	19(100)	*Fisher* = 0.490
Defervescence within 96 h, n(%)	22(100)		
Chest X-ray improvement, days, median (IQR)	5(3, 6)	5(4, 6.5)	0.464
Cough improvement, days, median (IQR)	3(2, 4)	3(2, 4)	0.083
Disappearance of wet rales, days, median (IQR)	4(4, 5.5)	4(3, 5)	0.128

Fisher’s exact test. DOX, doxycycline monotherapy; IQR, interquartile range; MIN, minocycline monotherapy

### Comparison of the clinical effectiveness of combined new-generation tetracyclines and glucocorticoid treatment

Patients who received minocycline or doxycycline combined with glucocorticoid were 53 and 50 in each case, and patients with fever in each group were 41 and 39, respectively. The defervescence rates within 24 and 48 h were higher in the DOXG group than in the MING group (92.3% vs. 83.3%, 100% vs. 92.7%, all *p* > 0.05). There were no statistically significant differences in the median time to improvement on chest imaging, median time to improvement of cough, or mean time to disappearance of wet rales (Table 3).

Nine patients with pleural effusion, eight with hypoxemia, and two with pulmonary atelectasis recovered after treatment with new-generation tetracyclines combined with glucocorticoids, and patients with hypoxemia recovered within 3 d.

**Table 3:** Comparison of the efficacy of new-generation tetracyclines combined with glucocorticoid therapy

Parameter	MING group	DOXG group	*p*-value
Number of patients, n(%)	53(51.5)	50(48.5)	
Defervescence within 24 h, n(%)	35(83.3)	36(92.3)	0.530
Defervescence within 48 h, n(%)	36(92.3)	39(100)	0.257
Defervescence within 72 h, n(%)	41(100)		
Chest X-ray improvement, days, median (IQR)	5 (4, 6.8)	6(5, 7)	0.213
Cough improvement, days, median (IQR)	3(2, 4)	3(2, 5)	0.620
Mean duration (days) of wet rales disappearance ± SD	3.52 ± 0.258	3.97 ± 0.291	0.266

IQR, interquartile range

### Analyzing the relationship between clinical course and fever duration before new-generation tetracycline treatment

In comparing the clinical course and fever duration before new-generation tetracycline administration in the 26 patients who developed pleural effusion, hypoxemia, pulmonary atelectasis, and greater than two-fold elevation of D-dimer levels at the time of therapy initiation with patients who did not develop these conditions, the Exp (B) corresponding to fever duration before new-generation tetracycline use was 1.318, and for each additional day of fever, the occurrence of pleural effusion, hypoxemia, pulmonary atelectasis, or greater than two-fold elevation of D-dimer complications was 1.318 times more likely (95% confidence interval [CI], 1.111–1.563; *p* = 0.001) (Table 4).

**Table 4:** Relationship between complications and clinical course and fever duration before new-generation tetracycline treatment

Complication	Number of patients, *n* (%)	Clinical course, days, median (IQR)	*P*-value	Fever duration, days, median (IQR)	*P*-value
Any complications			0.071		< 0.001
Yes	26 (15.8)	7 (6, 9)		6 (4.8, 7)	
No	139 (84.2)	6 (4, 8)		4 (3, 6)	
Pleural effusion			0.099		0.266
Yes	9 (5.5)	6.3 (5, 8)		5.2 (3.5, 6.5)	
No	156 (94.6)	6.6 (5, 8)		4.6 (3, 6)	
Hypoxemia			0.085		0.039
Yes	8 (4.9)	7.8 (6, 9)		6.5 (3.8, 7.8)	
No	157 (95.2)	6.5 (4.5, 8)		4.5 (3,6)	
> 2-Fold elevation in D-dimer level			0.954		0.004
Yes	12 (7.3)	7.5 (6, 9)		6.8 (5, 8)	
No	153 (92.7)	6.5 (4.5, 8)		4.4 (3, 6)	

IQR, interquartile range

### Risk factor analysis for defervescence time after treatment

To study the risk factors for fever reduction within 24 and 48 h after treatment with new-generation tetracyclines alone, age, sex, fever duration before treatment, duration of initial antibiotic, CRP, Procalcitonin, IL2R, IL6, LDH, ferritin, erythrocyte sedimentation rate (ESR), and D-dimer at the beginning of the disease were analyzed as a whole. Overall, disease CRP level and ESR were associated with fever resolution within 24 h (*z*=-2.132, *p* = 0.033; *z*=-2.156, *p* = 0.031), and age, initial CRP level and ESR were associated with fever resolution within 48 h(*t*=-2.727, *p* = 0.010;*z*=-2.237, *p* = 0.025; *z*=-2.479, *p* = 0.013). Statistically significant comparative differences were analyzed using a one-way binary logistic regression model, with defervescence within 24 h (1 yes, 2 no) as the dependent variable. The results showed that CRP and ESR at the early stage of the disease were correlated with a return to normal temperature within 24 h post-treatment (*p* = 0.020, *p* = 0.027). Significant factors from the one-way binary logistic regression analysis were then included in a multifactorial logistic regression. However, the final results did not identify any independent influences on the return to normal body temperature within 24 h after treatment with new-generation tetracyclines alone. Similarly, factors with statistically significant differences in the above comparisons were included in a one-way binary logistic regression model, using defervescence within 48 h (1 yes, 2 no) as the dependent variable. The results showed that ESR was an influencing factor (*p* = 0.022).

### Safety

No adverse drug reactions typically associated with tetracyclines, such as gastrointestinal disturbances, esophagitis, photosensitivity, tooth discoloration in children, and rare cases of hepatotoxicity, hypersensitivity, or idiopathic intracranial hypertension, were observed in this study.

## Discussion

MP is the smallest prokaryotic pathogen, and its pathogenic manifestations result from local tissue destruction, cytotoxicity, and host immune responses [11]. Eaton [12] originally isolated the first strain of MP in 1944 from a patient with atypical pneumonia. In 1970, Waites et al. [11] reported the first global isolation of a strain of MP from a 14-year-old patient who presented high resistance to erythromycin. Since the initial reports of widespread MRMP were first reported in Japan in 2004 [13], such cases have increased globally. The rate of macrolide resistance in China has also increased rapidly, and since the first report in 2005, there have been reports [4, 7] of 90– 100% MRMP in China.

In China, The prevalence of MP infection demonstrated a modest increase from June to July 2023, followed by a slight decline in August. With the commencement of the academic year, there has been an observed increase in the incidence of Mycoplasma pneumoniae infections in September [4]. A number of medical facilities have observed that the current outbreak of MPP infections is predominantly caused by MRMP. The mutation rate of the 23 S rRNA macrolide resistance gene in Beijing reached 97.1%, a figure that is considerably higher than the MRMP rate of up to 92.7% observed in hospitalized children during the 2021–2022 period [14]. In Shanghai, the prevalence of macrolide resistance among isolates increased from 83% during the period from 2005 to 2008 to 90% during the subsequent period from 2008 to 2009 [15].

From July to December 2023, the prevalence of macrolide-resistant Mycoplasma pneumoniae infection among children in Henan, China, reached 91%. The highest macrolide resistance rate was observed in the 6-10-year-old age group. The prevalence of macrolide resistance did not differ between male and female children [16]. This finding is consistent with the observation that the majority of cases in this study were concentrated in the 6-11-year-old age group (85.4%).

MRMP frequently presents with high fever, severe cough, and altered mental status, resulting in a prolonged course of illness, extended hospitalization, and an unfavorable prognosis. MRPP progresses rapidly and can progress to pneumonia within 2–3 days of a high fever. Chest X-rays or computed tomography scans typically reveal lobar pneumonia and solid lung lesions.

Macrolide resistance is a potential factor contributing to disease severity. As reported by Yan et al. [17], the percentage of patients diagnosed with severe MPP from 2013 to 2017 was 76.2% (48/63), 67.6% (23/34), and 60%. In 2019, the respective percentages were 0.7% (65/107), 73.7% (112/152), 79.1% (53/67), and 71.2% (301/423). Severe MPP can cause serious intra-and extrapulmonary complications. Intrapulmonary complications include plastic bronchitis, pleural effusion, pulmonary consolidation and necrosis, and pulmonary embolism; extrapulmonary complications include myocardial injury, liver function abnormalities, renal injury, anemia, encephalitis, and rash mucositis. Children are highly susceptible to life-threatening conditions such as diffuse alveolar hemorrhage, pulmonary embolism, which require significant medical resources [6, 18]. Therefore, treating eligible patients with sensitive anti-MP medications is important to improve treatment outcomes. In addition, appropriate antimicrobials are required to prevent MP community transmission.

MP lacks a cell wall, making it naturally resistant to β-lactams that target the cell wall. It is susceptible to drugs that inhibit protein synthesis (macrolides and tetracyclines) and DNA replication (tetracyclines and fluoroquinolones) [11]. Among these drugs, macrolides are considered the therapeutic choice in children, while tetracyclines and fluoroquinolones have age-related contraindications and are not recommended as first-line antibiotic choices.

In pediatrics, tetracyclines can be used for patients younger than 18 years when the benefits and risks are weighed and informed, which is also in line with the principles of the Chinese “Guidelines for the Diagnosis and Treatment of *Mycoplasma Pneumoniae* Pneumonia in Children (2023 Edition)“ [6]; that is, the new-generation tetracyclines, including doxycycline and minocycline, are alternatives for treating MPP, especially MRMP, and SMPP. Doxycycline and minocycline have similar chemical structures, bioavailability of 90– 100% after oral administration, and are well tolerated. To date, natural tetracycline resistance has not been reported in MP.

This study retrospectively analyzed the efficacy of oral treatment with new-generation tetracyclines for patients with severe MRMP pneumonia. The critical disease condition of patients is mainly due to MRMP infections, the use of new-generation tetracyclines and glucocorticoids aimed at targeting MRMP, and the overactive immune response. Clinical efficacy was remarkable, with no critical conditions or drug-related adverse reactions observed during treatment.

The therapeutic benefits observed in this study mainly included improvements in fever, imaging findings, disappearance of wet rales and cough improvement. Our analysis showed that, whether glucocorticoids were used in combination or not, the doxycycline group had a higher rate of fever reduction within 24 and 48 h compared to the minocycline group. Minocycline alone resulted in total fever reduction within 96 h, with a 48-h fever reduction rate of 63.64%, lower than the 85– 100% reported in the literature [19–22]. This may be due to all patients in this study having severe MRMP pneumonia. The defervescence rates of 63.2% within 24 h and 79.0% within 48 h after doxycycline treatment were in line with previous studies [20, 23], which reported a defervescence rate of 70– 87.6% within 48 h and complete defervescence within 72 h after doxycycline treatment [20, 24].

The most common point mutations in MRMP are A2063G and A2064G in structural domain V of 23 S rRNA, significantly increasing the minimal inhibitory concentration (MIC) of all macrolides [25, 26]. The MICs of erythromycin, clarithromycin, and azithromycin were significantly higher for MRMP, with the 90% MICs of erythromycin, clarithromycin, and azithromycin for MRMP with A2063 G (C or T) mutation being > 64 µɡ /mL, > 64 µg/mL, and 64 µg/mL, respectively. In contrast, the 90% MICs of minocycline and doxycycline were much lower at 1 µg/mL and 0.5 µg/mL, respectively [20], comparable to macrolide-sensitive strains [24, 27].This implies that new-generation tetracyclines are effective against both MRMP and macrolide-sensitive strains of MP, with their clinical therapeutic effects confirmed [21, 28].

Other studies [20, 21, 29] found that the MP DNA load of resistant strains was consistently higher than that of sensitive strains. The MIC of MRMP and DNA copy number changes before and after treatment were not monitored in this study due to the cost of treatment, necessitating further studies.

The pathogenesis of MP infection is associated with the production of community-acquired respiratory distress syndrome (CARDS) toxin, which induces the activation of NLRP3 (NLR-family, leucine-rich repeat protein 3) inflammatory vesicles, leading to the secretion of interleukin-1β and interleukin-18 by macrophages. This process induces hyperimmunization, compromises respiratory barrier integrity, and causes cellular damage [29]. Serum CRP [29] and ESR [30] are reliable predictors of overactive immune response. This is consistent with the regression analysis in this study, which found that CRP levels at the early stage of the disease and ESR were risk factors for fever remission time after treatment with new-generation tetracyclines alone. Based on the immunopathological mechanism of MP, combining tetracyclines with glucocorticoids can significantly improve clinical symptoms [31, 32], promote rapid improvement in clinical symptoms and chest imaging findings, and prevent disease deterioration and complications. The combination of new-generation tetracyclines and glucocorticoids in this study was beneficial for defervescence. The treatment success rate reached 100%, consistent with previous studies [32, 33].

The interaction between inflammation and the coagulation system in severe pneumonia can aggravate lung injury. In recent years, there have been reports of patients with MPP complicated by systemic arteriovenous thrombosis and even disseminated intravascular coagulation (DIC) [34, 35], which has a high rate of mortality and disability, resulting in more severe consequences than pneumonia. Previous studies [36] reported higher D-dimer levels in children with MPP compared to healthy children and even higher levels in children with severe MPP than in those with mild MPP, suggesting an excessive inflammatory response and vascular endothelial damage in this patient population. D-dimer levels were monitored in patients with severe MPP in our hospital, and the analysis revealed that the probability of complications like pleural effusion, hypoxemia, pulmonary atelectasis, or a > 2-fold elevation of D-dimer was 1.318 times higher for every 1-d increase in fever duration before new-generation tetracycline therapy (95% CI, 1.111–1.563; *p* = 0.001). The longer the duration of fever prior to tetracycline therapy, the greater the likelihood of hypoxemia (*p* = 0.039) and a greater than two-fold elevation of the D-dimer level (*p* = 0.004). This suggests that early treatment with new-generation tetracyclines is essential to avoid intrapulmonary and extrapulmonary systemic damage.

Owing to reports of permanent tooth discoloration and enamel hypoplasia in children treated with first-generation tetracyclines [37], it is recommended that tetracyclines be used only in children aged ≥ 8 years. Studies [24] indicate that tooth discoloration is influenced by factors such as treatment dose, duration of treatment, stage of tooth mineralization, and mineralization activity. However, data linking new-generation tetracyclines (minocycline and doxycycline) to tooth discoloration are limited. It has been reported [24] that short-term doxycycline treatment in children between the ages of 2 and 8 years either does not stain teeth or does so minimally. In addition, the American Academy of Pediatrics [23] recommends that doxycycline can be used for short periods (21 d or less) without age restriction because doxycycline has less calcium-binding activity than other tetracyclines and minimal risk of tooth staining with short-term use. Patients should still avoid excessive sun exposure, as doxycycline can cause photosensitivity. Other studies [38] found no significant difference in tooth discoloration and defects between children under 8 years who received oral minocycline for brucellosis and control groups. In our study, the duration of treatment with oral doxycycline or minocycline was 10 d, and no adverse effects associated with tetracyclines were observed.

This study still has certain limitations. First, this was a retrospective single-center study with potential selection bias. Second, the treatment effects were not compared across different age groups of patients. However, previous research [39] found no significant differences in clinical characteristics and rates of MRMP infections among preschoolers, school-aged children, adolescents, and adults. Finally, considering the limited cases of severe macrolide-resistant Mycoplasma pneumoniae pneumonia treated with azithromycin, We did not compare these two treatment strategies. It is crucial to undertake a multicenter, prospective clinical study in the future. This would not only help to verify the universality and reliability of the current findings but also increase the sample size, facilitating a more thorough comparative analysis.

## Conclusion

Treatment with new-generation tetracyclines was effective in children with severe MRMP pneumonia. Among these, doxycycline alone demonstrated a higher defervescence rate compared to minocycline alone within 24, 48, and 72 h compared with that of minocycline alone. Prompt treatment lowered the probability of pleural effusion, hypoxemia, pulmonary atelectasis, or D-dimer levels > 2 times the reference value. Short-term observations did not reveal any drug-related adverse effects. In future research, it is necessary to encompass a broader and more diverse patient population, with long-term follow-up data, in order to conduct a more comprehensive and thorough evaluation of treatment efficacy and safety.

## Data Availability

The datasets used and/or analyzed during the present study are available from the corresponding author on reasonable request.

## Abbreviations

CARDS: Community-acquired respiratory distress syndrome
CRP: C-reactive protein
ESR: Erythrocyte sedimentation rate
IL-2R: Interleukin 2 receptor
IL-6: Interleukin 6
LDH: Lactate dehydrogenase
MIC: Minimal inhibitory concentration
MP: Mycoplasma pneumoniae
MPP: Mycoplasma pneumoniae pneumonia
MRMP: Macrolide-resistant Mycoplasma pneumoniae
NLRP3: NLR-family, leucine-rich repeat protein 3
tNGS: Targeted next-generation sequencing
VAS: Visual analog scale
